# Supplementary material for: Comparison of the Phytochemical Variation of Non-Volatile Metabolites within Mother Tinctures of Arnica montana Prepared from Fresh and Dried Whole Plant Using UHPLC-HRMS Fingerprinting and Chemometric Analysis
Source: Molecules. 2022 Apr 24;27(9):2737. doi: 10.3390/molecules27092737 (PMC9103735; doi:10.3390/molecules27092737)
Supplement: Supplementary file 1 [file molecules-27-02737-s001.zip › molecules-1658904-supplementary.pdf]

Supplementary Materials

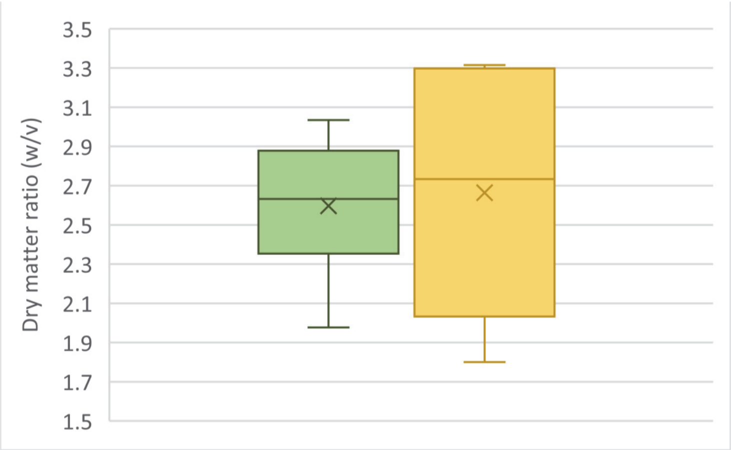

Figure S1. Mean dry residues ratio (in mg.mL^{-1}) obtained from mother tinctures. fMT: green box (n=12) and yellow box for dMT (n=12).

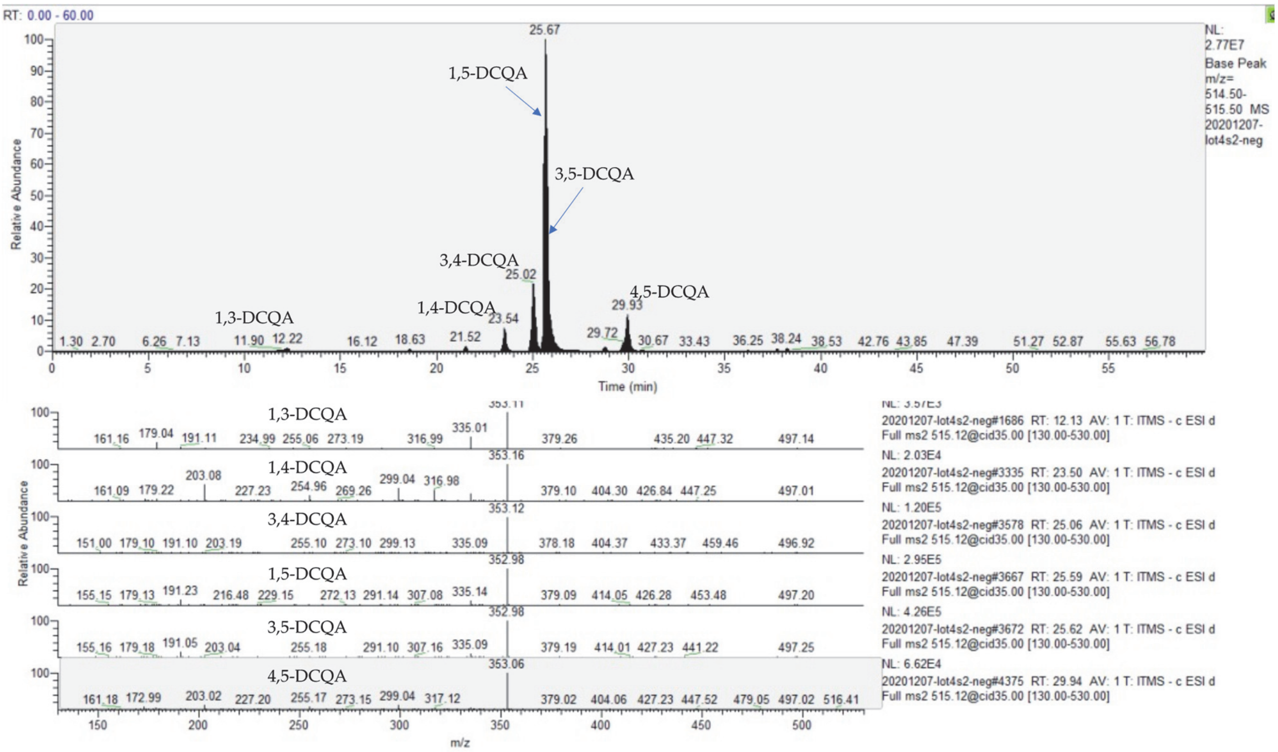

Figure S2. Selected ion chromatogram (m/z 515 (-)ESI) of fMT and MS/MS spectra of the 6 isomers of DCQA (m/z 515) showing differences in relative intensities of some product ions. The 1,5- and 3,5-DCQA are co-eluted at Tr 25.6 min.

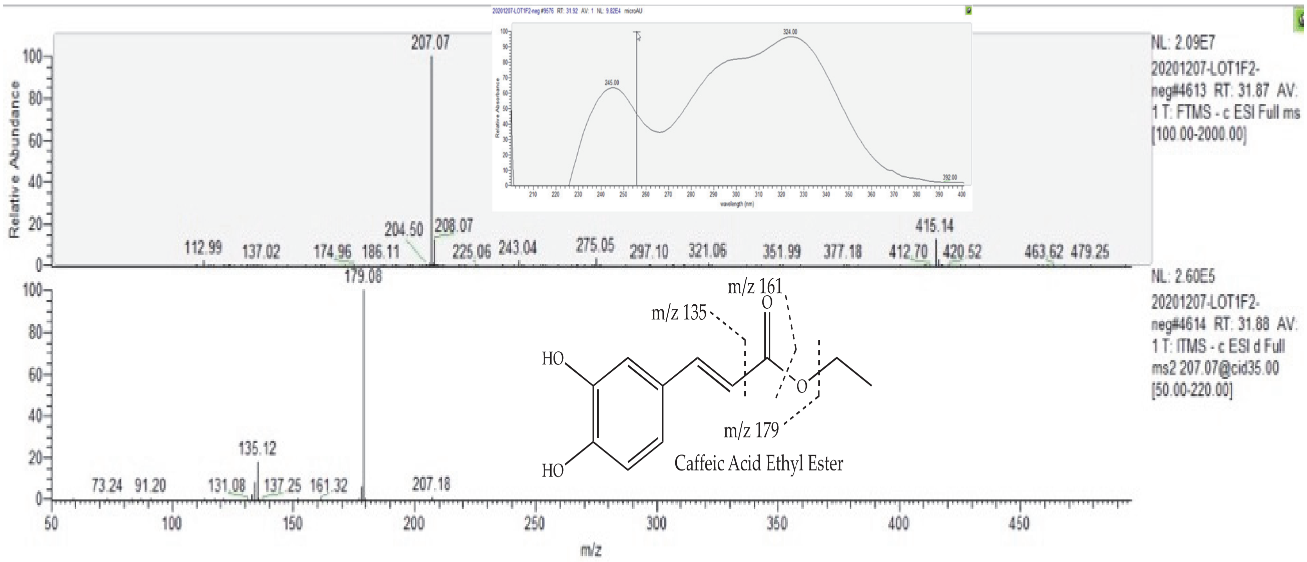

Figure S3. UV and negative ion ESI full MS spectra of the peak at 32.8 min (fMT) and MS/MS of the m/z 207 ion in coherence with the structure of Ethyl Caffeate as shown by the proposed structures of fragments.

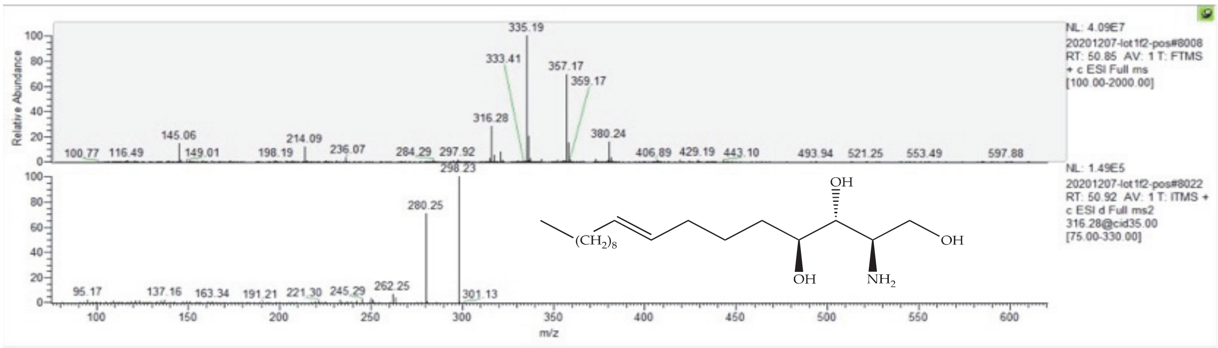

Figure S4. Full positive ion ESI MS spectrum of Rt 50.8 min peak and MS/MS spectrum of m/z 316 ion corresponding to Trihydroxysphingenine and showing the three successive losses of H_2O.

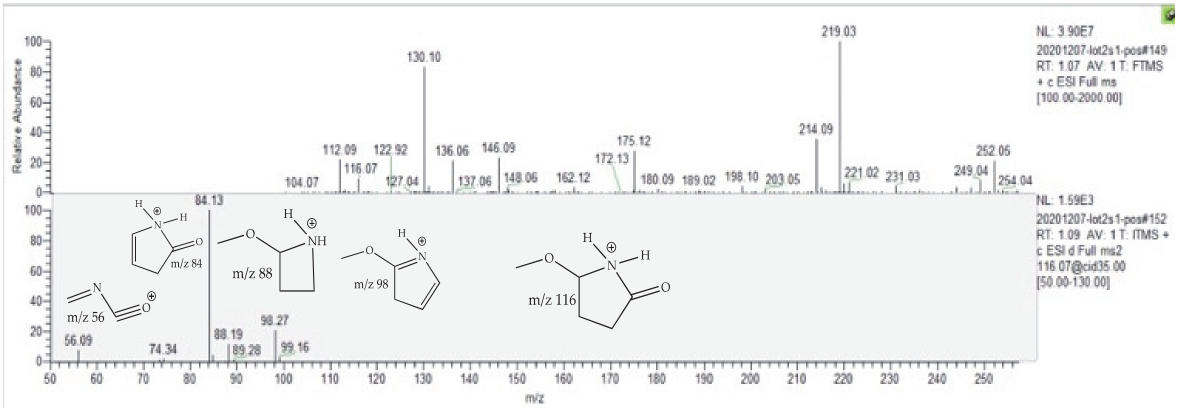

Figure S5. Full positive ion ESI MS spectrum of Rt 1.09 min peak and MS/MS spectrum of m/z 116 ion annotated to a pyrrolidinone Me ether derivative (with proposed structures for fragments).

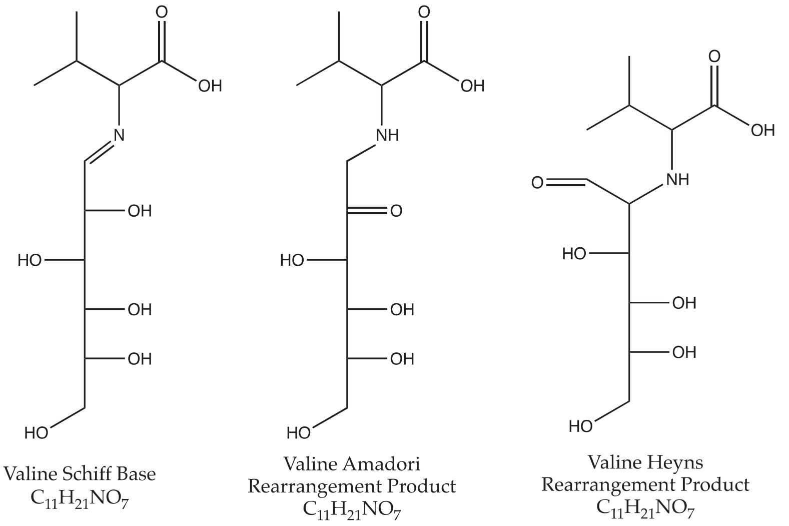

Figure S6. Structures of Valine Schiff base, Amadori and Heyns rearrangement products

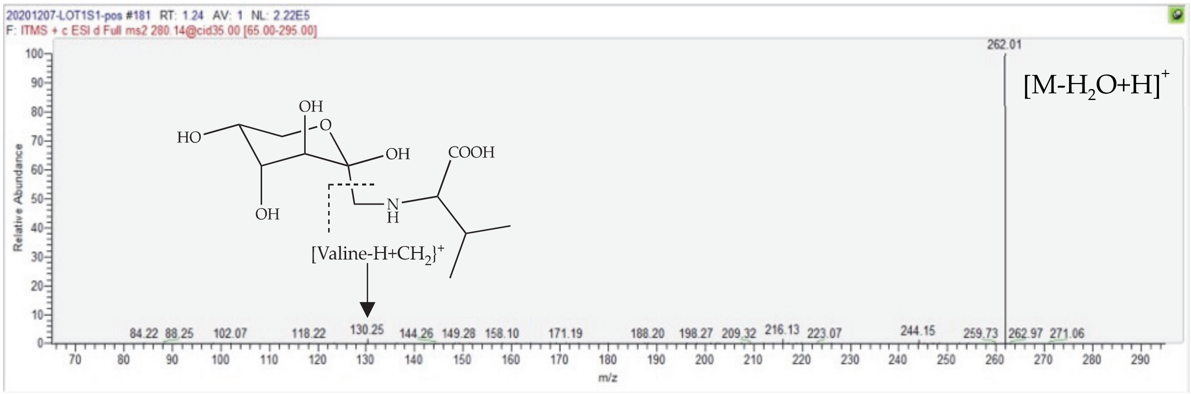

Figure S7. MS/MS spectrum of the protonated molecular positive ion of valine fructose conjugate at m/z 280 showing the characteristic [Amino Acid-H+CH_2]$^+$ ion of Amadori rearrangement products.

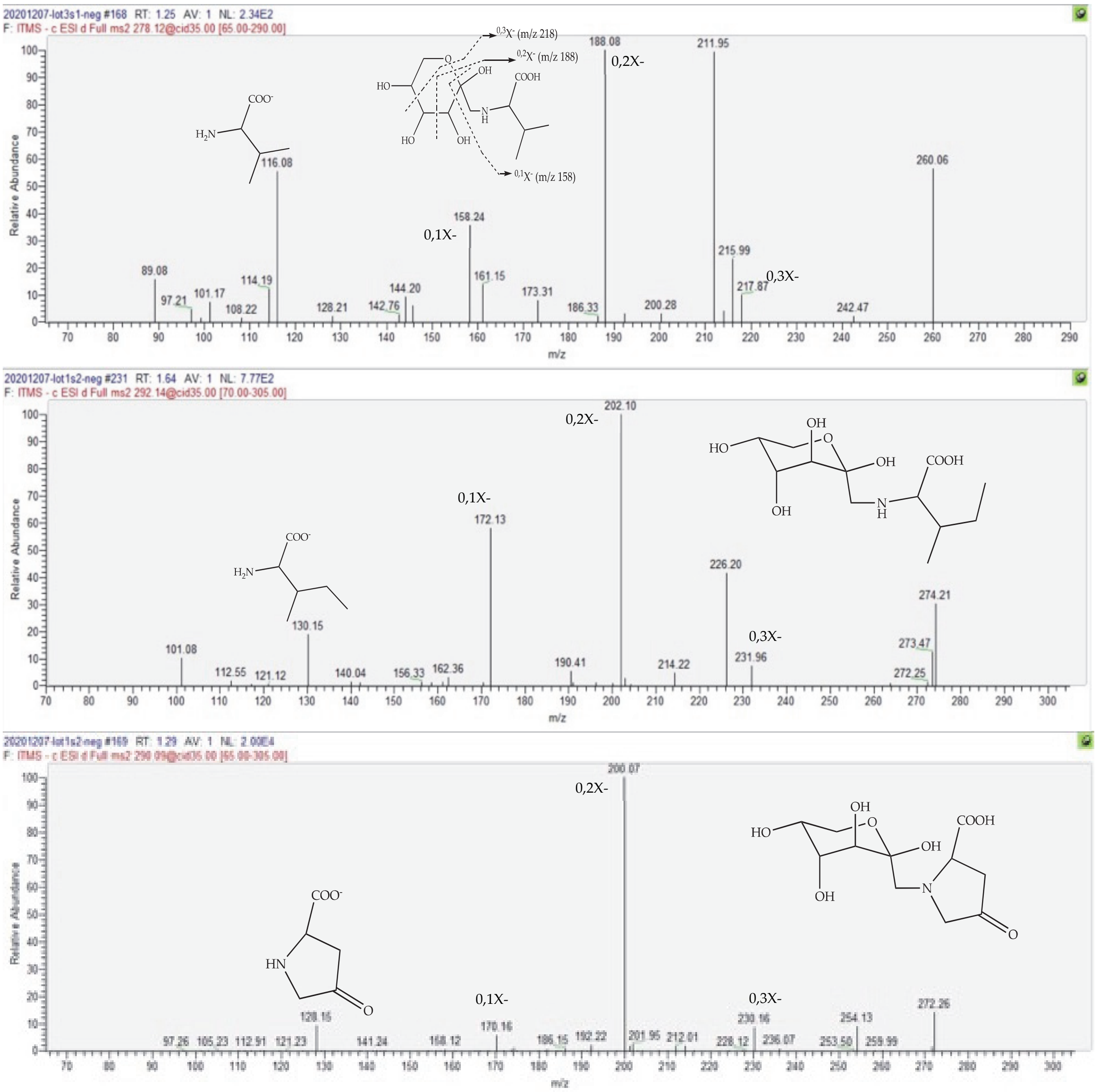

Figure S8. From top to bottom, negative ion MS/MS spectra of valine-, (iso)leucine- and oxyproline-fructose with diagnostic ions showing the similarities of fragmentation patterns between the three derivatives.

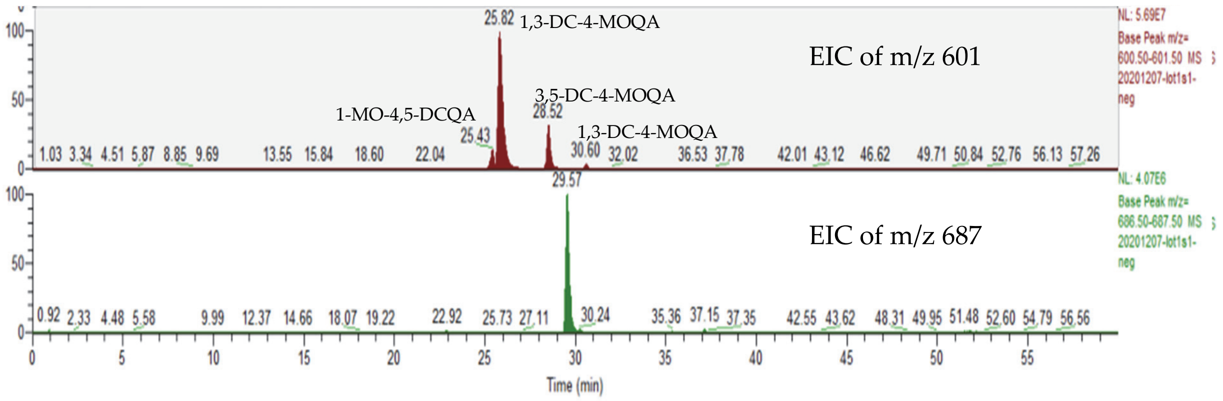

Figure S9. Extracted Ion Chromatograms (EIC) of the negative ions m/z 601 and m/z 687.

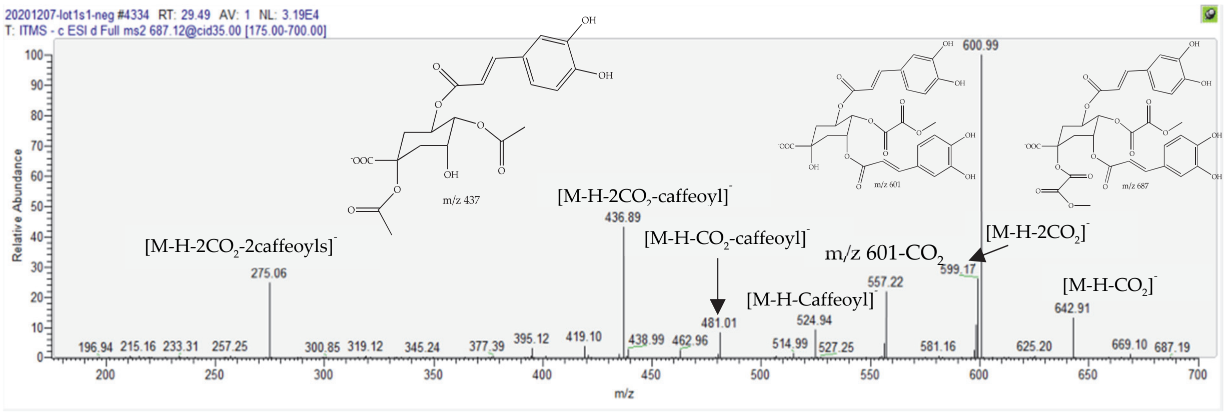

Figure S10. MS/MS spectrum of the [M-H]$^-$ ion at m/z 687 (putative 3,5-dicaffeoyl-1,4-dimethoxyoxaloyl-quinic acid) with suggested structures for the main product ions.

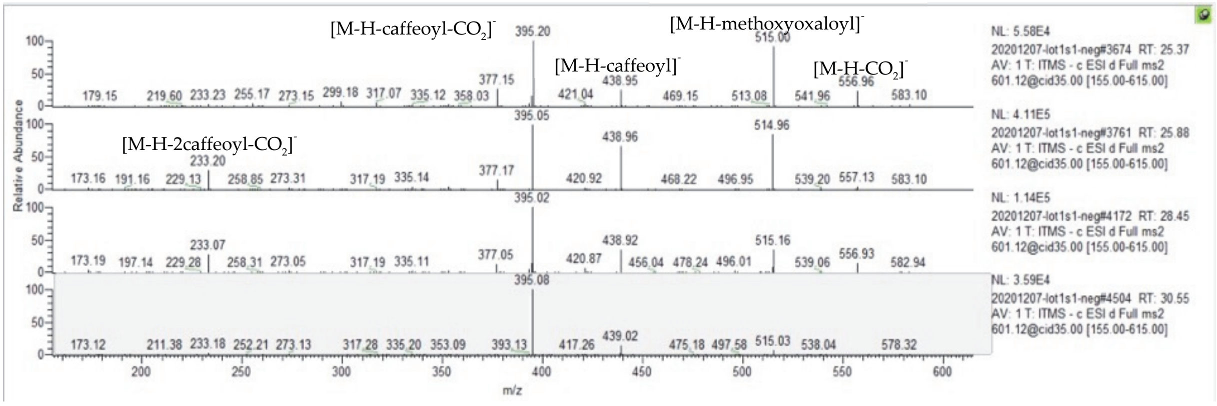

Figure S11. MS/MS spectra of the [M-H]$^-$ ions of the 4 regioisomers at m/z 601. Top to bottom: m/z 601 ions at Rt 25.4, 25.8, 28.5 and 30.6 min corresponding to 1-MO-4,5-DCQA, 1,5-DC-3-MOQA, 3,5-DC-4-MOQA and 1,3-DC-4-MOQA respectively.

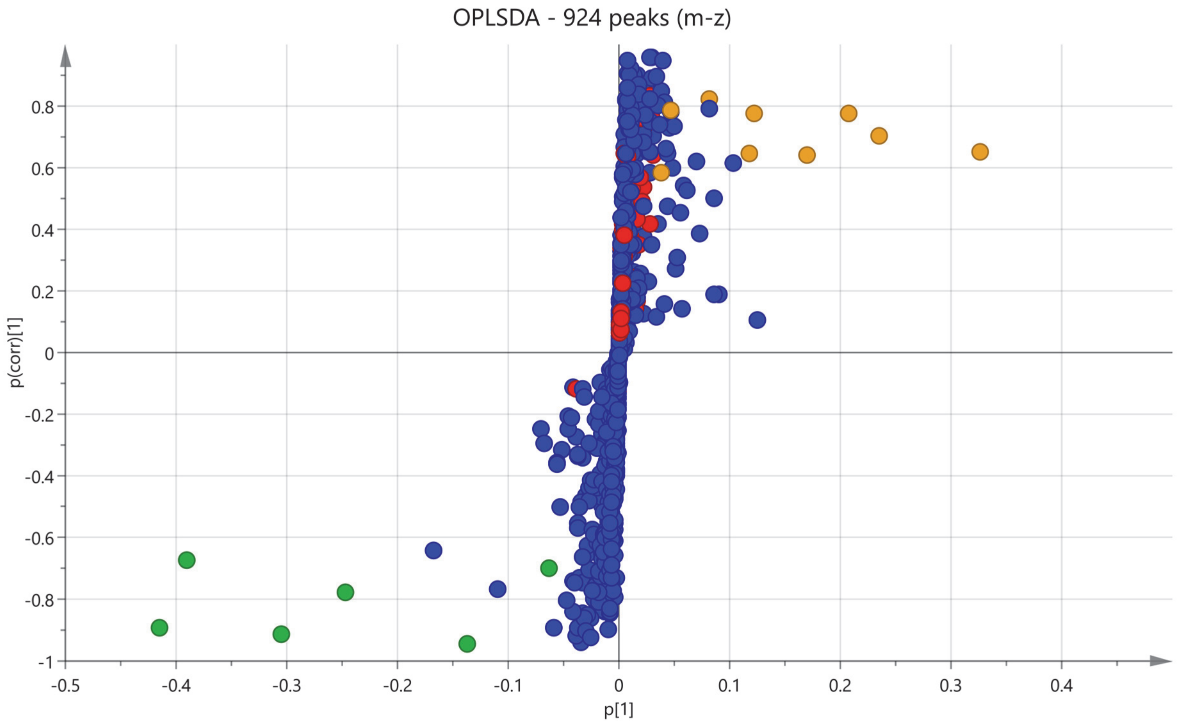

Figure S12. Multivariate analysis (OPLS-DA S-Plot) depicting the flavonoids (using MS-Finder annotation) in red and showing that they are more concentrated in dMT when compared to fMT but below the threshold set (p > |0.05|).

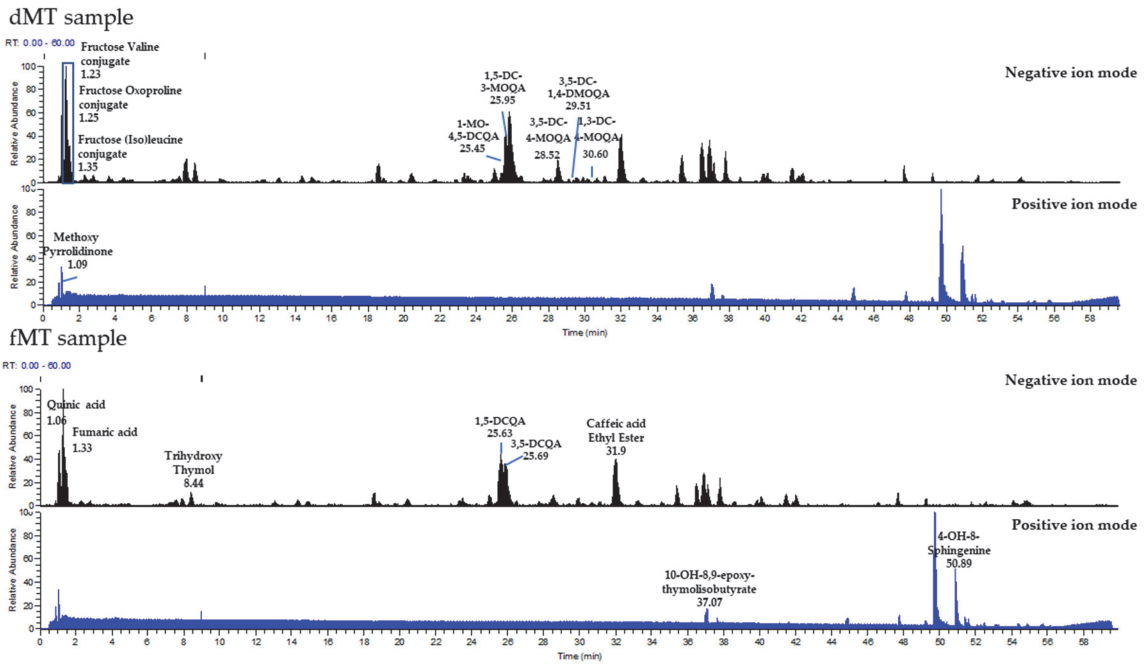

Figure S13. Positive and negative ion LC-MS base peak chromatograms of mother tinctures obtained from dried Arnica (dMT) and from fresh whole plant (fMT) showing the discriminant compounds.
